# Long-term tumour control of cystic and solid vestibular schwannoma treated with LINAC-based stereotactic radiosurgery: a retrospective analysis

**DOI:** 10.1007/s11060-025-05199-3

**Published:** 2025-08-11

**Authors:** Aaron Jin, Daniel Roos, Adrian Esterman, Sandy Patel, Peter Gorayski, Frank Saran, Ramkumar Govindaraj

**Affiliations:** 1 Radiation oncology department, Royal Adelaide Hospital, Adelaide, South Australia Australia; 2 Allied Health and Human Performance, University of South Australia, Adelaide, South Australia Australia; 3 Radiology department, Royal Adelaide Hospital, Adelaide, South Australia Australia; 4 University of South Australia, Adelaide, South Australia Australia

**Keywords:** Vestibular schwannoma, Acoustic neuroma, Stereotactic radiosurgery, Cystic, LINAC

## Abstract

**Purpose:**

The implications of intratumoural cystic change on management and treatment outcomes in vestibular schwannoma (VS) remain uncertain. This retrospective analysis aims to compare the treatment outcomes of solid and cystic tumours treated with linear accelerator (LINAC)-based stereotactic radiosurgery (SRS).

**Methods:**

Sixty-eight patients were analysed including 22 with cystic tumours. All received a marginal dose of 12 Gy to the 80% isodose line (range: 70–90%) delivered in a single fraction. Local progression was defined as a maximum linear dimension (MLD) enlargement of > 2 mm persisting for over 2 years, pseudoprogression as an MLD enlargement of > 2 mm within the first 2 years followed by stability or reduction.

**Results:**

With an average follow-up of 92.2 months, crude tumour control rates were similar: 95.5% for cystic and 93.5% for solid tumours. Pseudoprogression occurred in 7 patients (10.3%), 2 with solid (4.3%) and 5 with cystic VS (22.7%). The mean MLD reduction was 4.2 mm for solid tumours and 5.0 mm for cystic tumours (*p* = 0.51). Only pretreatment size correlated with the percentage reduction in tumour diameter (*p* = 0.025). Although a higher proportion of cystic tumours demonstrated at least a 10%, 20%, and 30% size reduction compared to solid tumours, none of these differences were statistically significant.

**Conclusion:**

LINAC-based SRS achieves similar tumour control for both cystic and solid VS. Despite cystic tumours being typically of higher Koos grade, they may demonstrate numerically greater reduction after SRS. Future studies should aim to standardise the criteria for classifying cystic tumours.

**Supplementary Information:**

The online version contains supplementary material available at 10.1007/s11060-025-05199-3.

## Introduction

Vestibular schwannomas (VS) are benign tumours arising from Schwann cells of the vestibular portion of the eighth cranial nerve. The incidence of these tumours has increased over the past few decades and is currently estimated to be between 19 and 33 per million per year [1–3]. The increase in the median age at diagnosis, and decrease in reported tumour size [2, 3] likely correspond to an increasing use of medical imaging and corresponding incidental detection of asymptomatic cases. Management approaches for VS include stereotactic radiosurgery (SRS), microsurgery (MS), and active surveillance. The use of active surveillance has increased in the management of these patients, as many tumours exhibit slow growth that can be monitored radiologically [4]. However, cystic VS pose a challenge in management and are often treated early due to their relatively rapid progression of symptoms and unpredictable growth patterns [5, 6].

The natural history and post-treatment course of cystic VS have garnered particular attention owing to their distinctive characteristics. These tumours are identified by hypointense regions on T1-weighted MRI and hyperintense areas on T2-weighted MRI, which differentiate them from homogeneous contrast-enhancing solid tumours. Although the pathogenesis of the cystic regions within these tumours is not fully understood, the enlargement of these cysts primarily contributes to their measured growth on serial imaging [5]. Early studies of SRS treatment for predominantly cystic tumours reported a higher rate of initial tumour enlargement following SRS, also primarily due to increased cyst size [7]. The incidence of cystic recurrences, often requiring surgical intervention, has also been observed to be more common in tumours that were cystic prior to treatment [8].

Concerns about cyst enlargement post-treatment have led to a preference for surgical management of cystic tumours, despite the reports of higher morbidity associated with surgical intervention [6]. One recent study reported inferior control rates in cystic compared to solid tumours treated with SRS [9]. However, the probability of local control of cystic tumours after SRS was similar to that of solid tumours in a meta-analysis of six studies [10].

The limited number of comparative studies examining outcomes between cystic and solid tumours does not provide a conclusive evidence base for SRS treatment, although most recent studies suggest similar outcomes for cystic and solid tumours. These studies are primarily retrospective cohort analyses with a small sample of cystic tumours, exclusively treated using Gamma Knife (GK) SRS and not all include long-term outcome data. Due to the rarity of these tumours, prospective studies directly comparing outcomes of cystic tumours treated with SRS or MS will not be feasible. Therefore, it is important to demonstrate SRS outcomes in cystic tumours by reporting data from diverse populations and settings to strengthen the evidence base. In this single-centre comparative analysis, we examine and report the long-term outcomes of cystic versus solid VS following LINAC-based SRS.

## Methods

Following approval from the institutional human research and ethics committee, we conducted a retrospective review of patients with VS treated at our institution between May 2006 and August 2021. Ninety-one patients with VS underwent SRS using a linear accelerator. According to institutional policy, patients were deemed eligible for SRS if they had a radiologically confirmed diagnosis of VS, exhibited growth on serial imaging, and were not suitable for MS or preferred SRS. Notably, Koos grade 4 tumours (brainstem displacement) did not preclude SRS, and the decision to proceed involved a multidisciplinary approach in collaboration with a neurosurgeon.

Treatment techniques have been previously reported [11, 12]. In brief, SRS was performed using stereotactic frame immobilisation. T1-weighted MRI and a planning CT scan were used to delineate the target volume. No PTV expansions were utilised. A dose of 12 Gy was prescribed to the 70–90% isodose curve. Following treatment, patients underwent MRI surveillance every 12 months for the first 3 years, then every two to three years thereafter, unless there was concern for progression.

Sixty-eight patients met the eligibility criteria for inclusion in the study. The following criteria led to the exclusion of 23 patients from analysis: neurofibromatosis type 2 (*n* = 2), inaccessible pretreatment MRI (*n* = 13), and follow-up imaging of less than 24 months (*n* = 8).

To ensure consistency, all pretreatment MRIs were reviewed by an experienced neuroradiologist to classify cases into cystic and solid groups. VS was classified as cystic if the maximum diameter of the T2-weighted hyperintensity was > 50% of the tumour’s largest cerebellopontine angle (CPA) diameter as shown in Online Resource 1. The tumours were measured along two axes: along the axis of the internal acoustic canal and perpendicular to it. The maximum linear dimension (MLD) was used as a pretreatment metric and for comparing post-treatment response.

Pseudoprogression was defined as MLD enlargement > 2 mm within the first 2 years, followed by stability or reduction. Local progression was defined as an MLD enlargement > 2 mm [13], either occurring beyond 2 years after treatment, or displaying persistent growth beyond this time. Treatment failure was defined as the need for additional treatment with either further SRS or surgery, indicated by consecutively enlarging tumours not classified as pseudoprogression, along with the presence of symptoms. Where available, pretreatment cranial nerve function (hearing, tinnitus, vertigo, trigeminal and facial neuropathy) was retrieved from patient records. Hearing was classified as serviceable or non-serviceable based on patient reporting or audiometric results. An inter-aural pure tone average of greater than 50 dB was considered non-serviceable. In cases of borderline audiometry, the patient’s reported serviceability was taken as final. We did not routinely perform speech discrimination.

The baseline characteristics of the solid and cystic tumours and treatment outcomes were compared using Fisher’s exact test or t-test for unequal variance. Due to the limited number of failures in each group, the percentage reduction in the MLD was used as the primary outcome. A Tobit regression model was used (with % reduction censored at 0) to evaluate the relationship between the predictors (age, gender, Koos grade, pretreatment size, maximum dose, prescription isodose line, and tumour classification - cystic versus solid) and the outcome variable. Tobit regression was used because the outcome—percentage tumour size reduction—is a continuous variable left-censored at 0%, with cases without MLD reduction or increase, recorded as 0%. Tobit models appropriately handle this form of censoring while preserving the continuous nature of the data above 0%. No upper censoring was applied, as the maximum observed reduction was 63%, reflecting the natural distribution of responses rather than a cap. This method allowed for unbiased estimation of associations despite the censored lower bound.

The percentage reduction was determined by comparing the size of the tumour in the pretreatment MRI and at the last follow-up. In theory, extended follow-up periods may result in greater tumour size reduction. Therefore, to account for differences in follow-up duration, logistic regression was used to assess the percentage of patients achieving 10%, 20%, and 30% reduction in tumour size post-treatment.

## Results

Twenty-two (32%) tumours were cystic. Cystic tumours were more common in females (Table 1) and generally larger, with 90.9% classified as Koos grade 3 or 4 compared with 67.4% of solid tumours. The mean axial pretreatment maximal tumour size in the cystic group, measured by MLD, did not differ statistically from that of solid tumours. All patients received a marginal dose of 12 Gy, with a median prescription isodose of 80% (range, 70–90%). The mean maximum dose to the tumour was 15.2 Gy (range 15.0–15.6 Gy).

**Table 1:** Baseline characteristics

	All patients (*N* = 68)	Cystic tumours (*N* = 22, 32%)	Solid tumours (*N* = 46, 68%)	*p* value
**Mean age (range)**	61.9 (SD 12.7)	66.2 (SD 12.4)	59.9 (SD 12.5)	0.057
**Sex**				
Male	32 (47%)	6 (27.3%)	26 (56.5)	0.037
Female	36 (53%)	16 (72.7%)	20 (43.5)	
**Treatment side**				
Right	34 (50%)	12 (54.6)	22 (47.8)	0.79
Left	34 (50%)	10 (45.4)	24 (52.2)	
**Koos grade**				
1	1 (1%)	0	1 (2.2)	0.049
2	16 (24%)	2 (9.1)	14 (30.4)	
3	26 (38%)	13 (59.1)	13 (28.3)	
4	25 (37%)	7 (31.8)	18 (39.1)	
Pretreatment mean size (SD), mm	20.0 (SD4.8)	21.0 (SD4.2)	19.5 (SD5.0)	0.20
**Mean Follow-up duration (SD) months**	92.2 (SD39.6)	82.7 (SD30.8)	96.7 (SD42.8)	0.13
**Median Follow-up duration (range) months**	86 (24–196)	77 (24–155)	87 (34–196)	

SD = standard deviation

The entire cohort had a crude tumour control rate of 94.1%, with similar rates for solid and cystic tumours (93.5% vs. 95.5% respectively). The mean follow-up period was 92.2 months (82.7 months for cystic and 96.7 months for solid tumours). Pseudoprogression occurred in 7 patients (10.3%) overall, 2 patients with solid (4.3%) and 5 with cystic VS (22.7%). Online Resource 2 presents the time trends in tumour size for patients experiencing pseudoprogression and progression. All cases of pseudoprogression were observed within 24 months following treatment, with subsequent reductions in tumour size occurring within 36 months. Cystic expansion occurred in 4 patients (18%). Tumour progression occurred in four patients: three with solid tumours and one with a cystic tumour (Table 2, Online Resource 2). Two patients had salvage surgery at 3 and 6 years post-treatment after 4 mm of tumour growth. The third patient was observed for 13 years due to age, during which the tumour increased by 5 mm; he died at 91 from unrelated medical issues. The fourth patient showed a 2 mm increase beyond 24 months but had no imaging after 43 months, so progression was presumed. Additionally, one solid tumour developed late cystic degeneration seven years after initial shrinkage and is now being monitored without intervention (Online Resource 3).

**Table 2:** Tumour response

Domain	*N*	Available for analysis	%	*p* value
**No progression**				
Overall	64	68	94.1	
Solid	43	46	93.5	1
Cystic	21	22	95.5	
**Pseudoprogression**				
Overall	7	68	10.3	
Solid	2	46	4.3	0.03
Cystic	5	22	22.7	
**Treatment failure**				
Overall	2	68	2.9	
Solid	1	46	2.2	0.55
Cystic	1	22	4.5	

The mean reduction in MLD was 4.2 mm (SD 4.0) for solid tumours and 5.0 mm (SD 4.9) for cystic tumours (*p* = 0.51). The sole predictor in the univariate analysis that correlated with the percentage reduction in tumour diameter was the pretreatment size (*p* = 0.025); age, gender, prescription isodose, Koos grade, and tumour characteristic (solid vs. cystic) did not show any correlation (Table 3).

**Table 3:** Univariate analysis of predictors of percentage reduction in maximum linear dimension

Variable	Category	B	SE (B)	95% CI (B)	*p* value*
Age		-0.03	0.16	-0.36–0.30	0.848
Gender	Male	1.00			
	Female	-6.24	4.02	-14.3–1.81	0.126
Isodose (%)		-1.09	4.02	-9.12–6.95	0.787
Maximum dose		1.76	15.44	-29.7–33.21	0.910
Side	Right	1.00			
	Left	-1.26	4.10	-9.45–6.94	0.761
Pretreatment size		0.85	0.42	0.00–1.69	0.049
Koos grade	IV	1.00			
	III	3.96	4.33	-4.71–12.6	0.364
	II	-6.87	5.32	-17.5–3.77	0.201
	I	-93.5	1973.6	-4040– 3860	0.962
Cystic	No	1.00			
	Yes	4.10	4.39	-4.68–12.88	0.354

*Based on Tobit regression

The likelihood of achieving a 10%, 20%, or 30% reduction in the MLD of cystic versus solid tumours, while acknowledging the difference in follow-up duration, did not show statistical significance (Table 4), although the relative shrinkage of cystic tumours was numerically greater in all three instances.

**Table 4:** Percentage reduction in MLD between cystic and solid tumours adjusted for follow-up duration

	Cystic (%)	Solid (%)	Odds Ratio	SE	*p* value
> 10% reduction	17 (89.5)	34 (81.0)	2.26	1.97	0.35
> 20% reduction	12 (63.2)	25 (59.5)	1.27	0.71	0.68
> 30% reduction	8 (42.1)	13 (31.0)	1.96	1.19	0.27

Baseline symptoms are presented in Table 5. Significant post-treatment cranial neuropathies were infrequent; five patients developed new or worsened cranial nerve symptoms, four of whom had Koos grade 4 tumours. Of these, three tumours were cystic (two Koos grade 4 and one Koos grade 3) and two were solid (Koos grade 4). Two patients (one had a cystic and the other a solid tumour) exhibited facial nerve neuropathy and were classified as House-Brackmann grade 5; neither patient regained facial function. These two patients also experienced trigeminal neuropathy. The three other patients (two with cystic tumours and the other with a solid tumour) had only trigeminal neuropathy. For all affected individuals, the trigeminal neuropathy remained permanent, and one patient with a cystic tumour also experienced neuralgia that required treatment with neuropathic agents.

One patient developed hydrocephalus within three months after treatment for a solid tumour, classified as Koos grade 4, with a maximum dimension of 30 mm and notable mass effect on the brainstem. The patient underwent shunt insertion, resulting in a full recovery. Six years after treatment, the tumour had decreased to 23 mm.

**Table 5:** Baseline symptoms

Symptom	*N*	Available for analysis	%
Serviceable hearing	28	63	44
Non-serviceable hearing	35	63	55
Vertigo	42	58	72
Tinnitus	32	41	78
Cranial nerve V symptoms	10	44	23
Cranial nerve VII symptoms	2	40	5

## Discussion

Our comparative analysis of treatment outcomes following LINAC-based SRS demonstrated no difference in tumour control rates between cystic and solid VS. Although cystic tumours exhibited numerically greater size reduction post-SRS, this did not translate into a significant difference in control rates. Our findings are consistent with studies on GK SRS comparing cystic and solid tumours [14–16].

The incidence of cystic tumours in our cohort was 32%, which is higher than that reported in some studies [5] but within the range described in others [10, 17]. The higher percentage of cystic tumours with Koos grades 3 and 4 is also concordant with other reports in the literature [9, 18].

We used a criterion of hyperintense regions in T2-weighted MRI that measured > 50% of the tumour diameter to differentiate cystic from solid tumours. The definition of cystic tumour varies significantly across studies (Online Resource 4), contributing to the wide variation in the reported incidence [10]. Studies have employed varying proportions of cystic versus solid areas to differentiate cystic and solid tumours. It ranges from 30 to 50% of the total volume or maximum diameter of the tumour, or the presence of cysts of any size observed in MRI [10, 15, 18]. We selected the 50% threshold as it is sufficiently large to measure accurately, thereby mitigating the risk of misinterpreting small variations in contrast enhancement as cystic changes. Nonetheless, this criterion may exclude tumours with smaller cysts that could represent early evolving changes in a cystic tumour. We did not differentiate cystic tumours based on the location or the nature of the cyst wall. Such classifications are more common in surgical series due to their relevance to surgical morbidity and the completeness of excision [19]. Currently, there is no conclusive evidence suggesting that these classifications have any significance for SRS treatment, provided that the entire tumour and cystic regions are included in the target volume. Particularly relevant to radiosurgery is the differentiation of cystic tumours by categorising them into micro and macrocystic types, as the latter may exhibit greater regression following SRS. We did not further categorise cystic tumours due to their limited number.

The pathogenesis of cystic change in VS is not fully understood, although several causes have been suggested: microhaemorrhages, necrosis, tissue degeneration, and increased expression of matrix metalloproteinases [5, 10, 19, 20]. Enlargement of cysts due to an osmotic effect, accumulating more fluid within the cyst, has also been proposed as a reason for the continued expansion of the cysts [5, 19]. The theory that cyst formation and enlargement are due to increased proliferation and cell growth lacks support since it has not been histologically proven [9, 19]. On the other hand, Ali et al. observed that among enlarging cystic tumours that were untreated, growth was mostly due to the cystic component [5].

The tumour control rate in our cohort was comparable to other studies including GK SRS series. The control rate for cystic tumours was 95.5% at an average follow-up of 83 months, which aligns with reported rates of 90–95% in the literature, most of which provide 5-year results [10]. In our cohort, there was no significant difference in the mean reduction in the size of solid versus cystic tumours, although the degree of post-SRS tumour shrinkage favoured cystic tumours numerically. Similar observations of a greater reduction in the size of cystic tumours compared to solid tumours have been reported [14, 15]. The selection of a minimum lesion diameter (MLD) threshold of 2 mm as a metric for reporting tumour progression aligns with precedents established in prior SRS studies [21, 22]. While volumetric reporting offers enhanced sensitivity, such measurements were not captured within this cohort; therefore, MLD was deemed an appropriate and practical metric that mirrors routine clinical practice. The > 2 mm criterion was chosen based on Kanzaki et al.’s consensus guidelines [13].

In this cohort, pseudoprogression was observed in 10% of cases and was more frequently seen in cystic tumours. Pseudoprogression is a recognised phenomenon following stereotactic radiosurgery (SRS) for vestibular schwannoma, though its underlying mechanisms have not been definitively established. Proposed mechanisms include osmotic fluid accumulation, vascular occlusion, acute inflammation, tumour necrosis, and chronic intra-tumoural haemorrhage [23–26]. The increased rates of pseudoprogression observed in this cystic group should be interpreted with caution due to the small sample size, but similar findings have been reported elsewhere [9, 15], with suggested mechanisms such as cystic expansion related to transudation from tumour wall vessels [27].

Pseudoprogression typically involves an initial increase in tumour size, followed by regression or stabilisation; however, the threshold and timing vary between studies. Definitions used in prior studies often cite a transient volume increase of > 10–30% [9, 14, 18, 28, 29], though some studies report any increase in size [15, 16, 30]. There is ongoing discussion regarding the timing of pseudoprogression in vestibular schwannoma. While many studies describe it as an early event occurring within 24 months post-treatment [9, 14], others report occurrences beyond 24 months [18, 29], which may indicate differing mechanisms for early versus late pseudoprogression.

In this cohort, pseudoprogression occurred within 24 months, with tumour regression noted within 36 months. These variations in time course emphasise the need for carefully timed clinical decision-making prior to intervention and highlight the importance of long-term follow-up. Differences in reporting criteria for both timing and threshold likely contribute to heterogeneity in incidence rates between studies.

Given the retrospective nature of this study, baseline symptom data were not available for all patients within the cohort. The reported high rates of symptoms at presentation likely reflect the clinician bias towards documenting positive symptoms and omitting them when absent. Patients were considered available for analysis only if symptom assessment was explicitly reported (either positively or negatively).

Patients with Koos grade 4 are often surgically managed due to concerns about post-SRS oedema causing worsening compression or cranial neuropathy. In our cohort, most patients who experienced new or worsened cranial neuropathy following SRS had Koos grade 4 tumours, including the patient who needed a shunt for post-treatment hydrocephalus. Frisch et al. [15] excluded these tumours from GK SRS. Conversely, Hasegawa et al. [21] found that mild brainstem compression did not affect treatment failure while severe compression, causing fourth ventricle deviation, did. Bowden et al. [14] treated Koos grade 4 tumours, achieving 92.9% control at 5 years, but noted all cases with facial neuropathy involved Koos grade IV tumours.

There are several limitations inherent to this study, beyond the bias associated with its retrospective nature and single-centre focus. We used maximum linear dimensions measured in the axial extra-canalicular plane, which may have inter-observer variations and are less accurate than volumetric analysis. We chose to use MLD since post-treatment volumetric assessment in diagnostic MRI is time-consuming and requires standardised techniques before it can be validly used [31]. Moreover, due to the small number of treatment failures, we examined percentage differences in axial MLD following SRS to determine if there were variations in response based on tumour characteristics. Sub-categorising cystic tumours into macro- and micro-cystic groups, was unfeasible due to the limited sample size.

## Conclusion

LINAC-based SRS achieves comparable long-term tumour control for both cystic and solid VS. In our series, cystic tumours tended to present with a higher Koos grade and demonstrated numerically greater size reductions following SRS. Careful consideration is required for Koos grade 4 cystic tumours due to potential post-SRS cyst enlargement and related morbidity. Future research should prioritise establishing standardised criteria for classifying cystic tumours and clinical applicability across studies.

## Supplementary Information

Below is the link to the electronic supplementary material.

Supplementary Material 1

## Data Availability

No datasets were generated or analysed during the current study.
