# Supplementary material for: Long-term tumour control of cystic and solid vestibular schwannoma treated with LINAC-based stereotactic radiosurgery: a retrospective analysis
Source: J Neurooncol. 2025 Aug 11;175(3):1155–61. doi: 10.1007/s11060-025-05199-3 (PMC12511140; doi:10.1007/s11060-025-05199-3)
Supplement: Supplementary file 1 — Supplementary Material 1 [file 11060_2025_5199_MOESM1_ESM.pdf]

Supplementary Information

Long-term tumour control of cystic and solid vestibular schwannoma treated with LINAC-based stereotactic radiosurgery: a retrospective analysis

Journal of Neuro-Oncology

Dr Aaron Jin
Radiation oncology department, Royal Adelaide Hospital, Adelaide, South Australia, Australia
Corresponding author e-mail: ajinradiation@gmail.com

Professor Daniel Roos
Radiation oncology department, Royal Adelaide Hospital, Adelaide, South Australia, Australia

Professor Adrian Esterman
Allied Health and Human Performance, University of South Australia, South Australia, Australia

Associate Professor Sandy Patel
Radiology department, Royal Adelaide Hospital, Adelaide, South Australia, Australia

Associate Professor Peter Gorayski
Radiation oncology department, Royal Adelaide Hospital, Adelaide, South Australia, Australia

Professor Frank Saran
Radiation oncology department, Royal Adelaide Hospital, Adelaide, South Australia, Australia
University of South Australia, Adelaide, South Australia, Australia

Dr Ramkumar Govindaraj
Radiation oncology department, Royal Adelaide Hospital, Adelaide, South Australia, Australia

Online Resource 1: Cystic vestibular schwannoma radiological classification

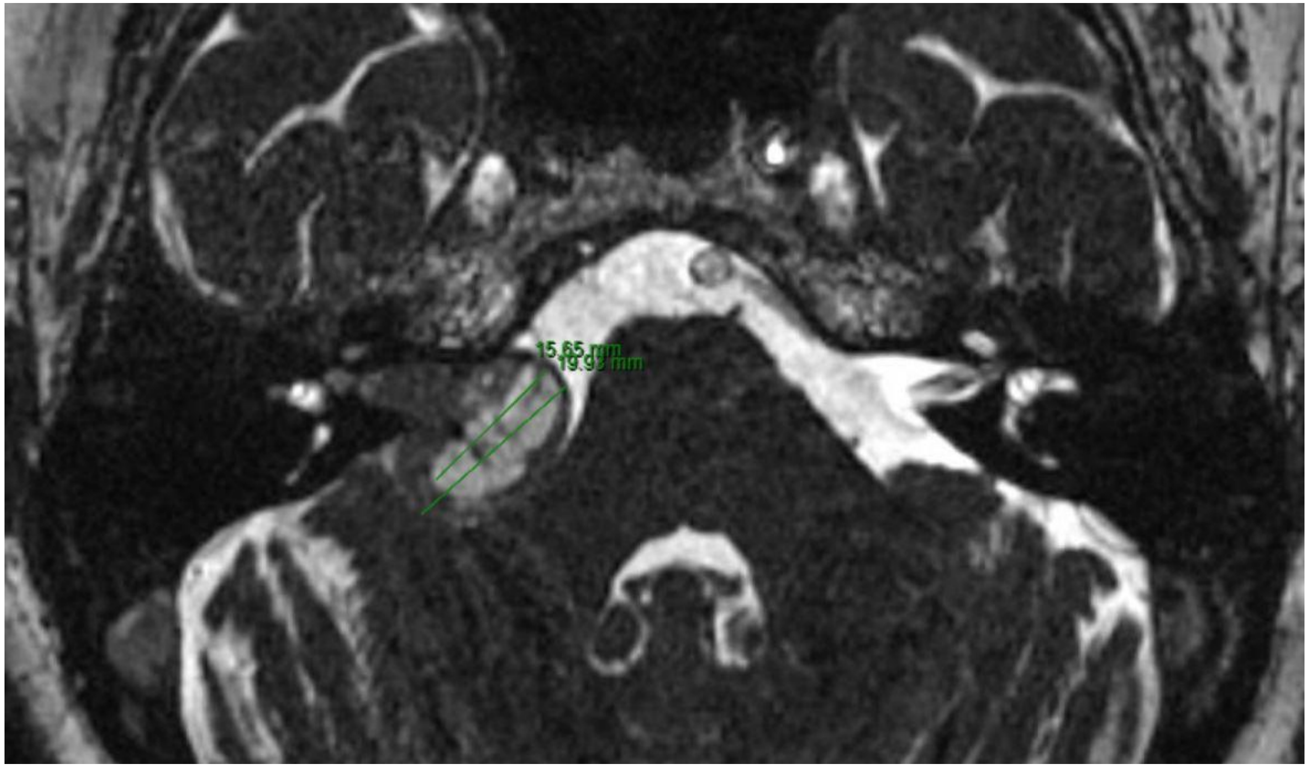

Figure 1: Cystic tumour with measurements illustrating the size of the cyst relative to the tumour diameter.

Online Resource 2: Tumour size trend for pseudoprogression and progression cases

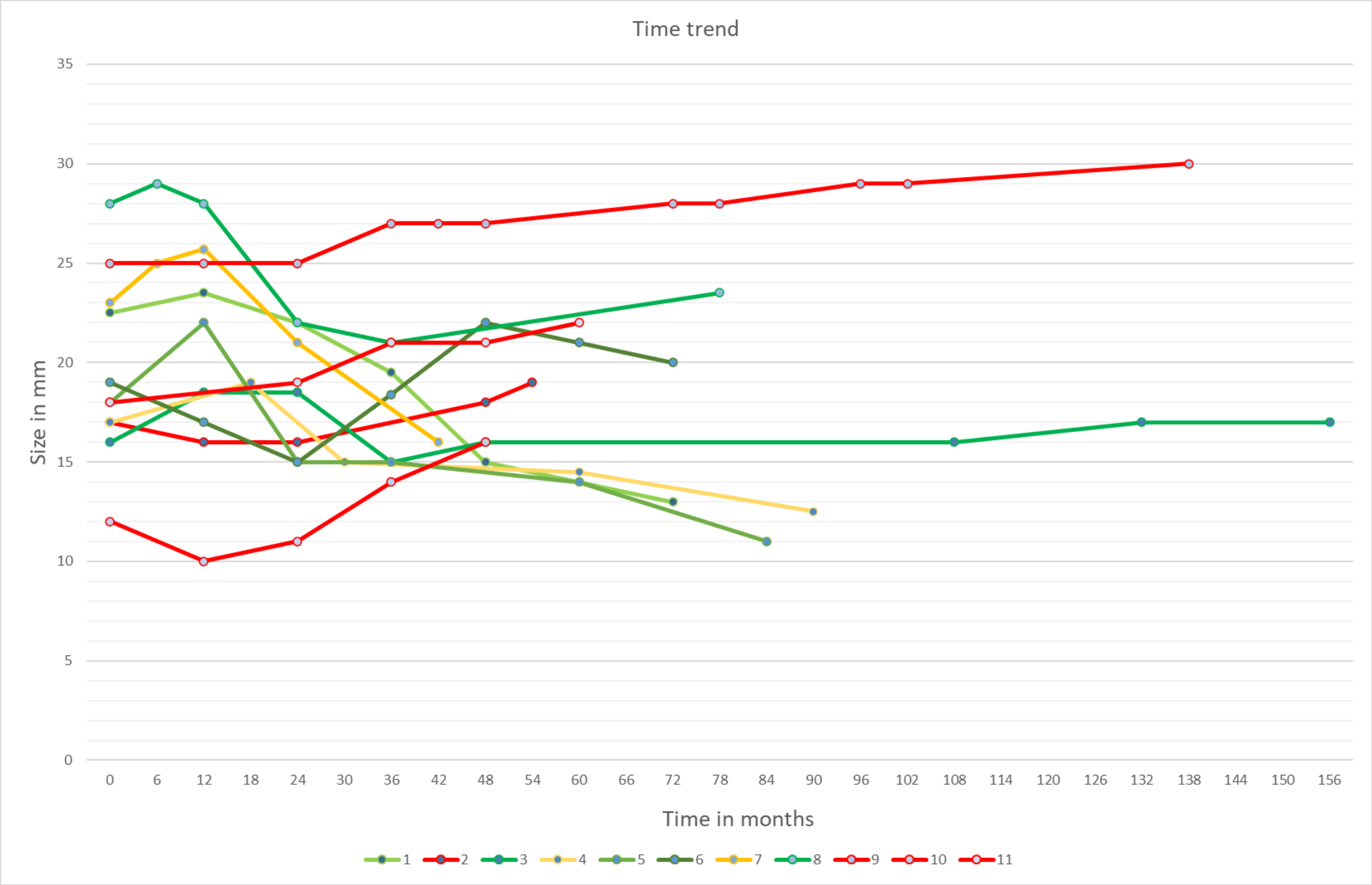

Time trends in tumour size for patients experiencing pseudoprogression (solid tumours represented in yellow lines and cystic tumours in green lines) and progression (red lines).

Online Resource 3: A case of late cystic degeneration

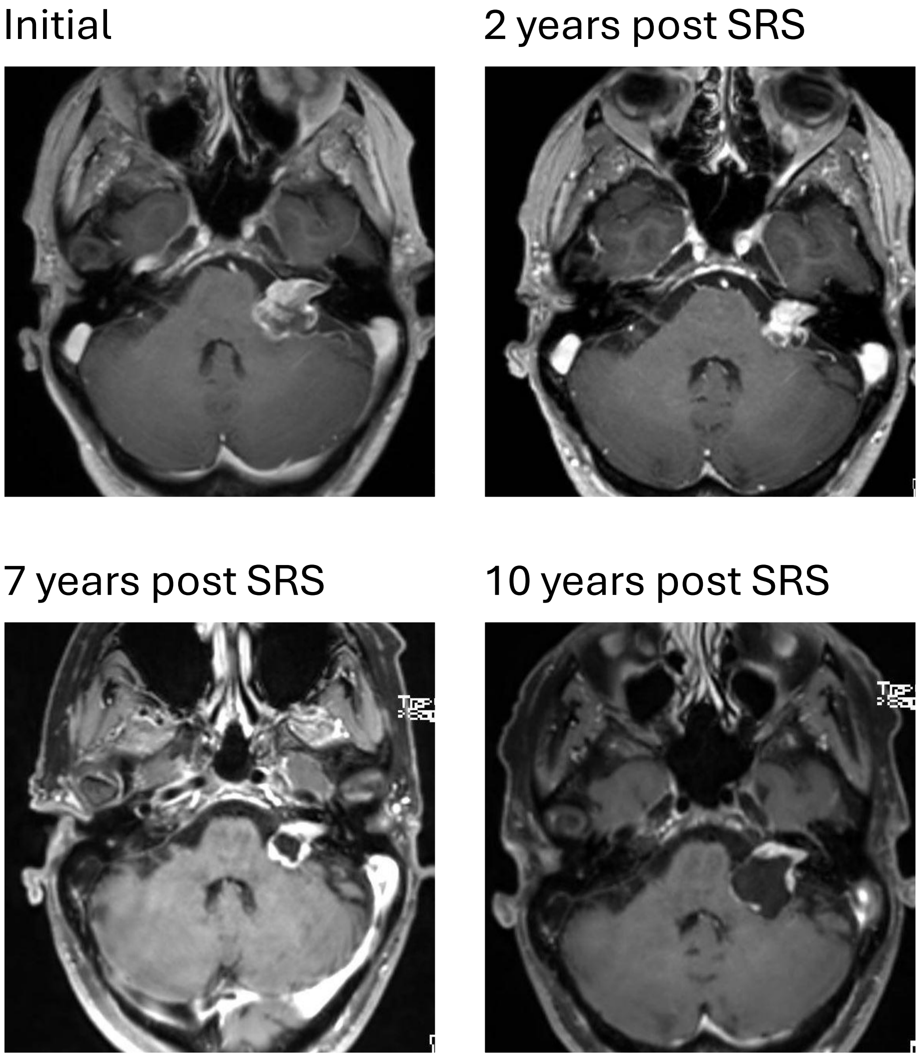

Late cystic degeneration observed after initial good response to treatment.

Online Resource 4: Gamma Knife radiosurgery trials for cystic vestibular schwannoma

Paper	Cystic classification	Progression definition	Pseudoprogression definition
Hasegawa et al. 2012 [21]	Classified as cystic and included mixed tumours with cystic components	Treatment failure: tumour enlargement (+2 mm) or radiation-induced peritumoural oedema requiring resection	Not reported
Klijn et al. 2016 [22]	Significant spherical or oval-shaped mass present that is T1-weighted hypointense and T2-weight hyperintense	Tumour progression: minimum diameter increase of 2 mm in any direction<br>Treatment failure: requirement for additional treatment with either secondary GKRS, microsurgery, or a combination of the 2 procedures	Not reported
Frisch et al. 2017 [15]	Total cystic diameter >50% of the maximum CPA axial tumour diameter	Tumour growth – no pre-defined metric	Not pre-defined, but reported 2 patients with significant cyst enlargement within 1 year, followed by spontaneous cyst shrinkage back to original pretreatment size
Bowden et al. 2017 [14]	Macrocystic: intratumoural cystic regions with T1-hypointensity and T2-hyperintensity<br>Microcystic: intratumoural cystic regions with T1-hypointensity and T2-hypointensity	Tumour growth: a volume increase of >10%<br>Tumour control: no need for additional interventions	>10% volume increase within 18 months
Wu et al. 2017 [28]	Cysts (including both intramural and extramural cysts) making up at least one-third of the whole tumour volume	Tumour progression: 10% increase in volume<br>Treatment failure: tumour enlargement (10% volume increase) at last follow-up	Transient tumour enlargement (10% volume increase) within the first postradiosurgical 12 months followed by either stability or regression

Lim et al. 2019 [16]	Method: containing dominant intratumoural or peritumoural cysts occupying >30% of the total tumour volume<br>Discussion: containing dominant intratumoural or peritumoural cysts occupying >30% of the total tumour diameter using greatest linear axial cerebellopontine angle dimension	Treatment failure: increase in lesion size after treatment at last follow-up MRI compared with the initial volume	Not reported, but excluded an increase in size within 1 year after treatment
Peker et al. 2022 [18]	Cyst wall exhibited contrast enhancement and cyst component accounted for >30% of the solid component on volumetric imaging	Progression: ≥20% increase volume<br>Tumour control: regression and stable disease	Transient tumour expansion: 10% volume increase followed by reduction to pre-GKRS size or less within 36 months post-GKRS
Wang et al. 2024 [9]	Presence of T2-hyperintense and gadolinium-contrast-negative cyst of any size	Early recurrence: tumour volume increase >30% persisting over 2 years after treatment	Transient tumour volume increase >30% within the first 2 years after SRS
